# Notes and News

**Published:** 1901-06-01

**Authors:** 


### NOTES AND NEWS.

Mr. Hugh H. Smiley, J.P., of Paisley, has handed over to trustees the sum of £5,000 to build and endow a cottage hospital for the town of Larne. He has nominated as the trustees Messrs. William Eccles, J. W. M'Ninch (solicitor), W. N. Brown, and his nephews, James Coey and Charles L. Mackean, and has entrusted to them the entire responsibility of selecting a site, erecting the building, and arranging details as to management, terms of admission, &c.

The ceremony of opening the new premises of Livingstone College at Leyton last week was performed by Mrs. Bruce, Dr. Livingstone's elder daughter. Each student before entering the college signs a declaration that he will not take to himself the title of "Medical Missionary," or assume the position of a qualified medical man. With these careful restrictions the college has secured the cordial co-operation of many members of the medical profession.

The Pinewood Sanatorium, now being erected near Wokingham by the London Open-air Sanatorium Association, is, we understand, organised on a financial basis somewhat similar to that of the Rowton Houses, namely—philanthropy at 2½ per cent. After the meeting held at Marlborough House a couple of years ago, at which the National Association for the Prevention of Consumption was inaugurated, the London partners of Messrs. Werner, Beit, and Co. agreed to find £20,000—the sum finally required will be nearer £30,000—to build and equip this sanatorium, on the terms that after all the expenses of running the establishment have been paid they shall receive 2½ per cent. for their money. Should the payments of the patients—which have been provisionally fixed at three guineas a head per week—yield any excess of income after payment of this interest, it will be devoted to the objects of the sanatorium in the manner which shall seem best to its committee. The institution stands on sandy soil, nearly 300 feet above sea-level, on a site which extends to 85 acres and is surrounded by pine-woods in every direction.

We understand that Mr. Alfred Harmsworth has given £10,000 to the London Hospital, Whitechapel, to endow one of the three installations which are in operation there for the "light cure" of lupus. The authorities of the hospital hope that this generous example will be followed, and that the other two installations, one of which was presented to the hospital by Queen Alexandra, may be similarly endowed, as each costs the hospital £400 a year.

Much interest is being shown in the scheme put forward by Earl Grey for the reform of public houses, which follows somewhat on the lines of the Gothenburg system which has apparently taken firm root in Sweden. A considerable number of meetings will be held during this month in furtherance of the movement, one at Lord Northbrook's house, another at Lord Stanhope's, and another in the course of the Archidiaconal Conference at Derby.

The Medical, Surgical, and Hygienic Exhibitors' Association has during this week held its fifth annual exposition of articles which it calls "professional exhibits"—all sorts and kinds of things interesting to those engaged in the treatment and prevention of disease. Of course, it is frankly and openly an advertising affair, but all the same it is a very good show. The public apparently loves to look at these things, but when one sees what one may call the mechanism of medicine displayed in all its nakedness—the pills, the physics, and the instruments by which sick man is helped back to such health as may be his fate—one cannot but feel a touch of sympathy with those who would say "get me out into the country and the sunshine and let me die in peace." There are still a few such—there used to be many—but most of us, nowadays, cling on to life even when it can only be maintained by the aid of the drugs and instruments here so daintily displayed. Strange taste!—and our work is to pander to it.

A case of much interest has recently been concluded at the Court of King's Bench, in which Lord Brougham claimed to recover damages from the defendants for breach of warrants in supplying him with linseed cake for his cattle which was alleged to be infected with anthrax bacilli, in consequence of which several valuable animals died. Professor McFadyean gave evidence that he tested the cake by pulverising it and mixing it with water, which liquid after straining was then injected into two guinea-pigs and a sheep. After several days one of the guinea-pigs was moribund, and after being killed, was found to be affected with anthrax. The liquid also was found to be swarming with anthrax bacilli. The other guinea-pig and the sheep showed no symptoms of anthrax. He admitted that it was possible that the cake might have been infected by the carelessness of persons who had touched the animals which had died, but was of opinion that the cake had not been so infected. Professor Boyce, Professor Sims Woodhead, Dr. Klein, and Professor Muir gave evidence that they had examined samples of the cake and had found no anthrax, and there was evidence that a large amount of the cake had been used on other farms without ill effect. Moreover, it was shown that anthrax had existed on the same farm, and also in the neighbourhood some years before. The plaintiff, however, got his case. The case well illustrates the value attached by jurymen to a single bit of positive as opposed to any quantity of negative evidence.

